# Resistance to receptor tyrosine kinase inhibitors in solid tumors: can we improve the cancer fighting strategy by blocking autophagy?

**DOI:** 10.1186/s12935-016-0341-2

**Published:** 2016-08-02

**Authors:** Sanja Aveic, Gian Paolo Tonini

**Affiliations:** Neuroblastoma Laboratory, Pediatric Research Institute-Città della Speranza, Padua, Italy

**Keywords:** Receptor tyrosine kinase inhibitors, Autophagy, Drug resistance, Combination treatment

## Abstract

A growing field of evidence suggests the involvement of oncogenic receptor tyrosine kinases (RTKs) in the transformation of malignant cells. Constitutive and abnormal activation of RTKs may occur in tumors either through hyperactivation of mutated RTKs or via functional upregulation by RTK-coding gene amplification. In several types of cancer prognosis and therapeutic responses were found to be associated with deregulated activation of one or more RTKs. Therefore, targeting various RTKs remains a significant challenge in the treatment of patients with diverse malignancies. However, a frequent issue with the use of RTK inhibitors is drug resistance. Autophagy activation during treatment with RTK inhibitors has been commonly observed as an obstacle to more efficacious therapy and has been associated with the limited efficacy of RTK inhibitors. In the present review, we discuss autophagy activation after the administration of RTK inhibitors and summarize the achievements of combination RTK/autophagy inhibitor therapy in overcoming the reported resistance to RTK inhibitors in a growing number of cancers.

## Background

### Intersection between receptor tyrosine kinases and autophagy

Receptor tyrosine kinases (RTKs) are transmembrane glycoproteins that participate in the transduction of external signals into cells [1]. Due to their intrinsic enzymatic activity, RTKs transmit a cascade of reactive phosphorylation events after interacting with extracellular signaling molecules, leading to cell growth, migration, differentiation, survival or apoptosis. The final response depends on the nature of the received signal [2]. Under physiological conditions, the release of growth factors or other extracellular ligands from cells, and hence their binding to RTKs, is strictly regulated and well balanced [3]. In general, once the signal is received, cross-linking of neighboring RTKs is required for message propagation in terms of the phosphorylation cascade. This step enables the autophosphorylation of tyrosine residues on RTKs, which stimulates the kinase activity of the RTK itself. Afterwards, the phosphate groups are transferred from the ATP to tyrosine residues of RTK-docking proteins in the cytoplasmic interface [4]. Phosphorylated tyrosine residues, which enhance the enzymatic activity of RTKs, can be recognized by several cytoplasmic proteins with Src homology-2 (SH2) or phosphotyrosine-binding (PTB) domains. In this manner, multiple receptor tyrosine residues became phosphorylated, and the signal is transduced, thereby triggering different signaling cascades. The two principal intracellular protein pathways triggered by RTK activation are the mitogen-activated protein (MAP) kinase RAS/RAF/MEK/ERK and the phosphatidylinositol 3-kinase (PI3K)/AKT/mTOR pathways [5].

**Fig. 1:**
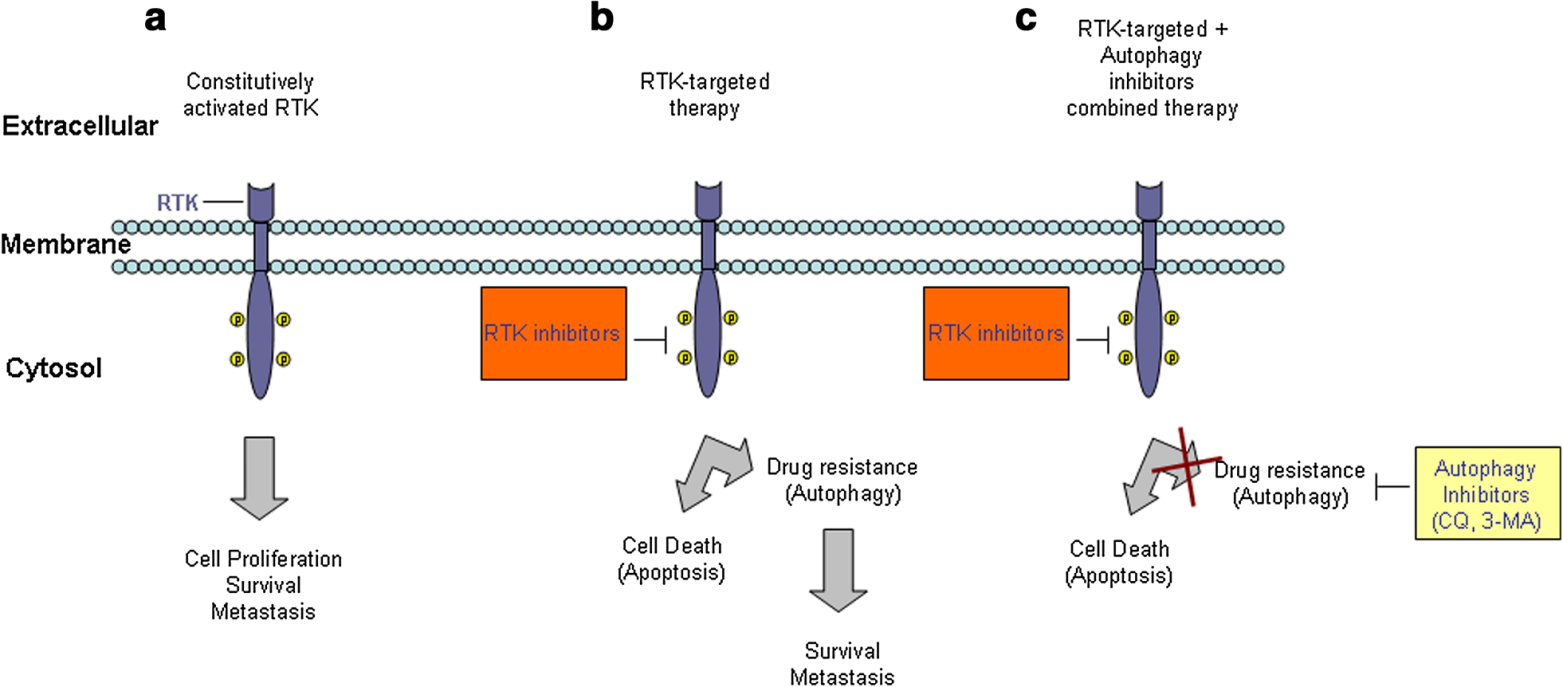
RTK activation in malignant cells and possibility for the combined therapy. **a** The constitutive, ligand-independent, catalytic activation of RTK under pathophysiological conditions leads to uncontrolled cell proliferation as well as increased cell survival and metastasis. **b** Blocking RTK function with inhibitors (*orange* *blocks*) activates a bypass mechanism that involves the induction of autophagy, which may contribute to the acquisition of drug resistance. **c** The enhanced efficacy of combination approaches (RTK inhibitors and autophagy inhibitors, such as chloroquine (CQ) or 3-methyladenine (3-MA); *yellow* *blocks*) with respect to a single RTK-targeted strategy suggests this method as more promising strategy for the elimination of tumor cells

Because the main role of RTKs is to regulate cell growth and survival, it is not surprising that their abnormal activity has been correlated with tumor development and progression [6]. The constitutive, ligand-independent, catalytic activation of RTKs in pathological conditions (Fig. 1a) may arise from chromosomal rearrangements that comprise RTK genes and/or from point mutations or amplification of RTK genes [7, 8]. The involvement of dysregulated RTK-dependent signaling in cellular transformation justifies the rational for the development of RTK antagonists and their inclusion in targeted cancer therapy. However, the most recent RTK-targeted therapy failed to improve the cure rate because the malignant cells activate defense mechanisms and acquire resistance [9]. One mechanism that might sustain the drug resistance of tumor cells is autophagy [10, 11].

**Fig. 2:**
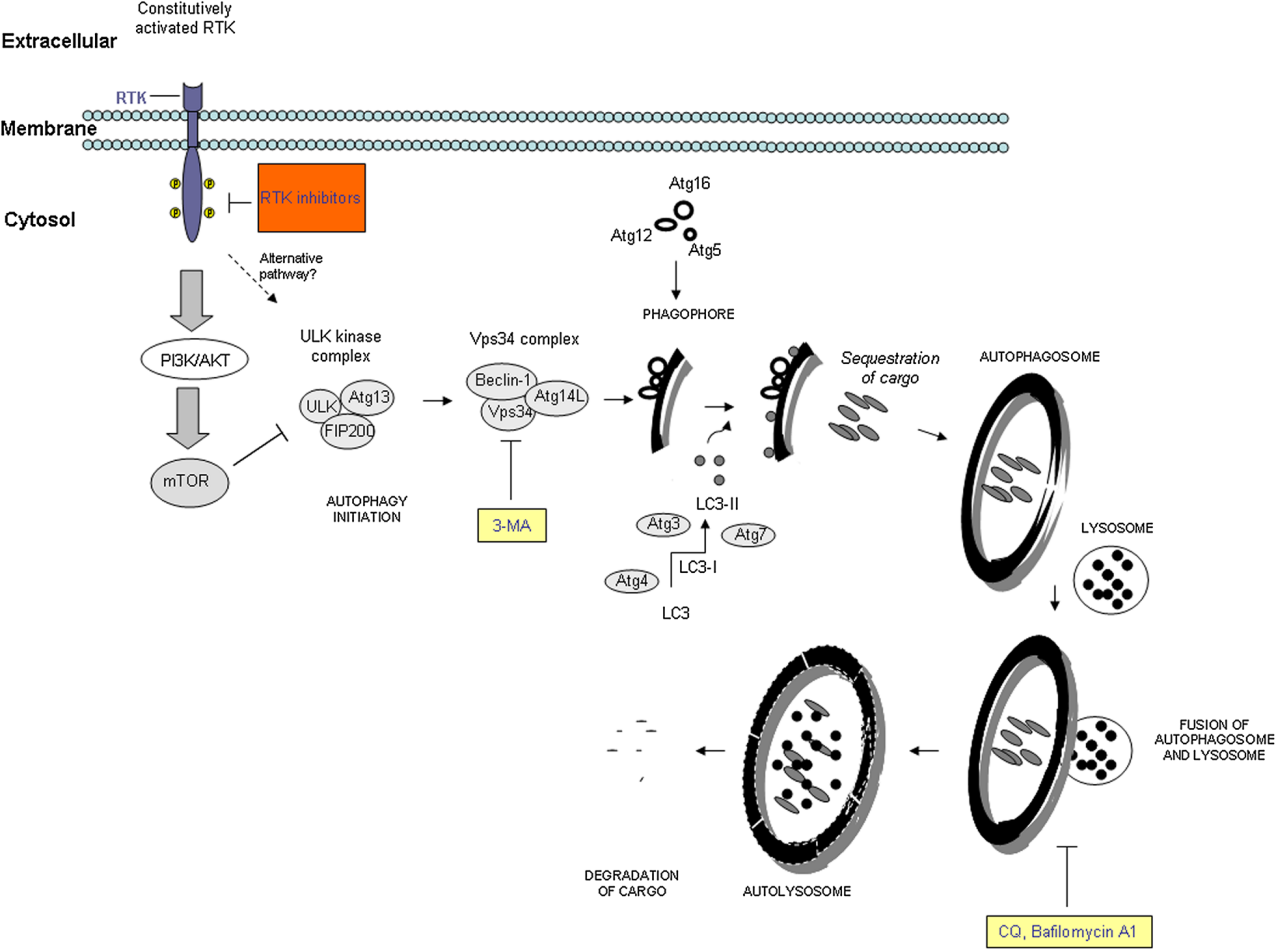
Potential molecular mechanism of autophagy induction in the presence of RTK inhibitors. One of the pathways through which autophagy could be induced after RTK inhibition is PI3K/AKT/mTOR, which, in addition to other roles, blocks the initiation of autophagy. The initial step of autophagy is regulated by the ULK kinase complex, which is comprised of ULK, ATG13, and FIP200. This step is followed by the alignment of additional proteins that form the Vps34 complex (Vps34, Beclin-1, and Atg14L), which is needed for phagophore creation. Afterwards, the phagophore progresses toward the autophagosome by recruiting other proteins (Atg12/Atg5/Atg16 and LC3) within the double-layered membrane. At this step, as the autophagosomal membrane is increasing in size, the cytosolic contents are engulfed, thereby filling up the autophagosome with degradable intracellular cargo. Completely matured autophagosomes then fuse to lysosomes, generating autolysosomes, which enables the degradation of the included cargo. In this way, diverse cellular molecules are recycled and made available for reuse by the cell. Additional bypass mechanisms that may lead to the induction of autophagy after the addition of RTK inhibitors are not excluded (interrupted* arrow*). *Orange and yellow blocks* represent RTK and inhibitors of autophagy, respectively

Autophagy is an important catabolic process that regulates the degradation and recycling of organelles and proteins within the cell. It participates in the regulation of general cellular homeostasis [12]. Once autophagy is activated, a phagophore is formed; then, membrane nucleation occurs, creating a double-membraned autophagosome (Fig. 2). The autophagosome carries the cell’s cytoplasmic cargo and organelles, and then fuses to the lysosome; ultimately, the vesicular content is degraded and reprocessed [13]. Currently, many proteins have been identified as regulators of autophagy, and their interactions have been confirmed to be crucial for the processing of autophagic vacuoles. Apical regulation of autophagy is accomplished by the formation of the Unc-51-like kinase 1 (ULK1)/autophagy-related (Atg) 13 complex [14, 15], which then interacts with Beclin-1/Vps34 complex and subsequently recruits additional Atg proteins (Atg 3, Atg 4, Atg 5, Atg 7, and Atg 12) during the generation of an autophagosome [16]. Then, the processing of microtubule-associated protein 1 light chain 3 (LC3), a well-accepted hallmark of autophagosomes, starts with cleavage in the cytosol (LC3-I) and lipidation (LC3-II) [17, 18], guiding the autophagosome toward an autophagolysosome. Another multifunctional adaptor protein, p62/SQSTM1, is likely responsible for the specificity of the autophagosome-targeting process and links the ubiquitinated proteins for degradation by lysosome [19, 20]. Numerous scientific reports have highlighted the association between unbalanced regulation of autophagy and cancer [21, 22]. Moreover, autophagy stimulation has been associated with tumor resistance to RTK inhibitors (Fig. 1b).

The present review aims to discuss autophagy activation as a possible mechanism involved in impeding the cytotoxicity of RTK inhibitors. It will summarize troublesome resistance as frequent manifestation that arises when RTK inhibitors are used to treat different malignancies. Furthermore, it will postulate a rational for the use of a combination therapeutic strategy with autophagy inhibitors and RTK inhibitors to improve their success.

### Molecular mechanisms of RTK inhibitors induced autophagy

Recent years have brought in evidence numerous reports that survey effectiveness of RTK inhibitors in the treatment of solid tumors. Initial enthusiasm for the RTK inhibitory treatment as principal targeted therapy waned when patients started to develop resistance to these inhibitors [23]. At molecular level, several mechanisms have been described along with acquired resistance, among which are secondary mutations, and activation of compensatory pro-survival signaling pathways [24]. One of the protective mechanisms that lately emerges along the use of RTK inhibitors is autophagy. Several signaling pathways triggered after activation of RTKs are also known regulators of autophagic process [25]. Therefore, it is not surprising that RTKs inhibition can have direct consequence over autophagy regulation.

The PI3K/AKT/mTOR is one of the most important signaling pathways that regulate autophagy [26], and at the same time represents one of downstream pathways activated by RTKs. Hence, inhibition of RTKs strikes the axis of PI3K/AKT/mTOR signaling directly, causing down-regulation of PI3K/AKT/mTOR proteins. Elimination of mTOR as a negative regulator of autophagy allows in following its activation (Fig. 2). Being protein kinase itself, mTOR is considered as a principal inhibitor of autophagy in mammal cells [27]. It acts not only as negative regulatory factor of autophagy, but also as a controller of cellular metabolism, which makes mTOR a key node in the regulatory network of cell homeostasis. In tumor cells, mTOR expression is frequently deregulated [28]. For that reason, several studies are concentrated on understanding the precise role of mTOR in cancer, and uncovering whether mTOR might be an interesting druggable target and under which circumstances [29].

### MicroRNA and autophagy

Ultimately, the studies that indicate the microRNAs (miRNAs) as the important intermediary of autophagy regulation in the eukaryotic cells are flourishing [30]. These ~22 nt long, non-coding, endogenous RNAs regulate negatively the expression of genes related to numerous cell processes including autophagy. By binding to the 3′ untranslated region (UTR) of the target messenger RNAs, miRNAs cause their degradation and inhibition of translation [31]. After defining miR-30a as the first miRNA able to down-regulate Beclin-1 [32], and hence impact autophagic activity, the number of miRNAs connected with the regulation of core autophagy controllers is constantly growing [33]. These evidences indicated for a direct connection between miRNAs and autophagy and opened a new frame of studies confirming the extreme complexity of autophagy regulation. Knowing that autophagy can impact sensitivity of cancer cells to RTK inhibitors, it can be expected that miRNAs are somehow involved in this regulation as well. Indeed, the correlation between miRNAs’ expression and resistance to some RTK inhibitors has already been reported in lung cancer by Garofalo and collaborators [34]. However, the interplay between autophagy, miRNAs and resistance to RTK inhibitors is still insufficiently explored. Evidently, we need more data to conclude whether or not the modulation of the specific miRNAs, by miRNA mimetics or inhibitors, could omit autophagy stimulation provoked by RTK inhibitors and prove more successful therapy.

### Deregulated RTKs in solid tumors and their inhibitors

*EGFR* epidermal growth factor receptor, also known as ErbB1 [35], was the first RTK to be discovered, and it has played an important role in connecting RTKs to cancer. EGFR was recognized as a possible anticancer target in the mid-1980s [36], but it was introduced in clinical oncology much later. Ever since, particular benefits from targeting EGFR have been observed for patients with advanced non-small cell lung cancer (NSCLC) [37, 38]. The small molecule inhibitors erlotinib (Tarceva, Genentech) and gefitinib (Iressa, AstraZeneca) are the two most commonly EGFR inhibitors [39]. Beside NSCLC, these inhibitors have been used to treat patients with pancreatic cancer [40], and they are currently used for lung adenocarcinoma therapy (ClinicalTrials.gov Identifier: NCT02155465). Many additional EGFR inhibitors have been tested in vitro, and several other malignancies with deregulated EGFR function have been identified. In many of the studied tumors, autophagy activation emerged as a recurrent problem during the administration of EGFR inhibitors [41]. This observation suggests that autophagy may be involved in the poor response to certain drugs or in the acquisition of drug resistance. Additionally, it implies that inhibiting autophagy may be a possible therapeutic strategy for overcoming resistance to RTK inhibitors [42].

Sugita and colleagues [43] reported that gefitinib caused a strong induction of autophagy in the NSCLC cell line PC-9. Importantly, when they blocked autophagy with clarithromycin, an antibiotic known to block autophagic flux [44], they observed marked induction of cell death. In a separate study, increased autophagy was reported in gefitinib-resistant PC-9 cells, whose survival was successfully impaired when gefitinib was combined with 3-methyladenine (3-MA) or chloroquine (CQ) [45], two potent autophagic inhibitors. Han and colleagues [41] reported similar findings indicating that autophagy was responsible for the impaired sensitivity of cancer cells to either gefitinib or erlotinib. Hence, when the pharmacological or genetic inhibition of autophagy was combined with RTK inhibitors, the cytotoxic effects of these drugs were notably improved. Based on the reports on NSCLC and autophagy, it is rational to suggest that inhibiting autophagy could be a promising therapeutic strategy for enhancing the efficacy of current EGFR-targeted therapy.

The efficacy of gefitinib in blocking the growth of breast cancer cells has also been tested. In these cells, the formation of autophagosomes was observed as an early event after treatment [42]. The alteration in autophagic flux was confirmed by studying the expression of p62 and LC3, and was dependent on drug concentration. A decrease in LC3-II protein levels was observed when autophagy was abrogated by 3-MA or bafilomycin A1. Due to the inhibition of autophagy, increased apoptosis was observed in these cells. AG1478 (Tyrphostin AG-1478), another EGFR inhibitor, was connected to the induction of autophagy after being administered to ovarian cancer cells in vitro. In this case, the protective role of autophagy in response to EGFR inhibition was largely diminished by 3-MA, which reduced metastasis occurrence in vivo [46]. Erlotinib is also capable of stimulating autophagy in lung adenocarcinoma, but this action can be dampened by co-treatment with CQ. In fact, use of erlotinib and CQ successfully suppressed tumor growth in a xenograft mouse model in a synergistic manner [47, 48]. Moreover, erlotinib-acquired resistance was shown to be autophagy dependent in lung adenocarcinoma, likely through LC3A activation, which favored tumor cell survival and proliferation. In contrast, the inhibition of autophagy by siRNA or CQ reverted these pro-survival effects, making the cells sensitive to erlotinib [49].

Conversely, an additional body of evidence has connected EGFR (ErbB1)-targeted treatments with the stimulation of autophagy, thereby helping tumor cells’ death escape [50]. Similar findings were reported after targeting another member of the ErbB family, Her2 (ErbB2). In particular, ErbB2 overexpression has been found in breast cancer patients classified within the poor survival group. In these patients, trastuzumab (Herceptin) was used to target ErbB2 [51], but unfortunately, drug resistance was encountered during treatment. To solve the problem with resistance, trastuzumab was administered in combination with CQ, which successfully improved the efficacy of the RTK inhibitor. The same combination strategy was used in the generation of trastuzumab-sensitive breast cancer cells with primary resistance to this inhibitor. Cufí et al. [52] showed a strong synergy between trastuzumab and CQ; this combination affected the survival of trastuzumab-refractory cells and thus reduced tumor growth. Altogether, these findings highlight the frequent activation of autophagy after the administration of RTK inhibitors against the ErbB family members ErbB1 and ErbB2, and provide evidence that autophagy might be one of the principal mechanisms responsible for drug resistance.

*VEGFR, PDGFR, and c-Kit* Many RTK inhibitors have more than one target. Sunitinib malate (Pfizer; sunitinib in following), a small molecule inhibitor, targets vascular endothelial growth factor receptor (VEGFR), platelet-derived growth factor receptor (PDGFR), and stem cell tyrosine kinase receptor (c-Kit). After being implicated in the development of diverse malignancies, these RTKs have been targeted with sunitinib in metastatic renal cell carcinoma, imatinib-resistant gastrointestinal tumors, and pancreatic neuroendocrine tumors [53]. Importantly, resistance was observed in renal and colon cancer cells during prolonged in vitro treatment with sunitinib [54]. In fact, a study on renal and colon cells demonstrated the involvement of acidic lysosome formation in the intracellular distribution of sunitinib. More precisely, the sequestration of sunitinib by lysosomes was a substantial event in resistant cells, which became sensitive to this drug after co-treatment with bafilomycin A1 or ammonium chloride. This observation implied that abrogating lysosomal function is a potential solution for negating the adaptive resistance to sunitinib. Curiously, the extent to which autophagy protects cells from sunitinib in this in vitro experimental model system remains unknown. On the other hand, sunitinib showed good cytotoxic activity when tested in breast, cervical, colorectal, hepatocellular, laryngeal, and prostate cancer cell lines. More importantly, in many of these cells, CQ was a good choice for combination treatment with sunitinib because it increased the effects obtained with the RTK inhibitor alone [55].

Sorafenib (Nexavar) is an inhibitor of the VEGFR, c-kit, PDGFR, and Raf/MEK/ERK signaling pathway [56]. It has been considered as a treatment for diverse malignancies, including advanced hepatocellular carcinoma (HCC) [57]. Nevertheless, complete tumor regression is not always observed in patients treated with sorafenib. One of the mechanisms for the partial resistance to sorafenib in HCC is autophagy. The induction of autophagy was confirmed in hepatoma cells based on LC3B processing, a clear decrease of p62 protein levels, and an intense accumulation of autophagosomes [58]. Characteristic cytostatic effects caused by sorafenib were largely improved when this drug was combined with pharmacological inhibitors of autophagy (CQ or bafilomycin A1), which successfully activated apoptosis in hepatoma cells. Similar findings have been reported by Yuan et al. [59].

*MET* also known as c-Met or hepatocyte growth factor receptor (HGFR), is an RTK with deregulated function in certain types of cancer. Aberrant activation of MET during oncogenesis may occur due to *MET* gene overexpression or activating point mutations [60]. PHA665752 (Pfizer) and EMD1214063 (Merck), two c-Met inhibitors, are currently under preclinical and clinical investigation, respectively, for the treatment of gastric cancer. In vitro, these inhibitors induced autophagy in gastric cells, resulting in increased *LC3B* and *ATG7* mRNA levels. When they were used in combination with autophagic inhibitors (3-MA or CQ), a negative impact on cancer cell viability was achieved. The experimental evidence obtained from the use of MET inhibitors has suggested that the cyto-protective effect gained by activating autophagy might be a serious obstacle for effective therapy. Therefore, inhibiting autophagy could be an approach to guarantee significant improvements in the therapeutic strategy of inhibiting RTKs [61].

*ALK* Anaplastic lymphoma kinase dysfunction has been reported in several solid tumors, including neuroblastoma (NB). Its continuous activation in NB was reported to stem from point mutations or amplification of the *ALK* gene [62]. Recently, we tested entrectinib (Ignyta), an anti-ALK, anti-ROS1, and anti-Trk compound, for its ability to impair NB cell growth and proliferation [63]. We found a correlation between the level of the drug’s reduced potency and autophagy activation, which was cell type specific. As expected, inhibiting autophagy with CQ improved the compound’s activity and increased NB cell death. Although ALK inhibition and autophagy have not been extensively studied until now our findings, and those from Ji and colleagues [64] in lung cancer, suggest that ALK inhibition might provoke autophagy-dependent resistance. In the same study, crizotinib (Pfizer), an ALK, MET and ROS1 inhibitor, was used to generate resistant lung cancer cell lines. It was noted that the down-regulation of ALK protein was associated with the induction of autophagy, thus showing cyto-protective features. When autophagy was inhibited by CQ, the sensitivity of drug-resistant lung cells to crizotinib was restored, once again providing a rationale for targeting autophagy when there is evidence of resistance to RTK inhibitors.

Altogether, the data summarized herein suggest that RTK inhibitors frequently cause the induction of autophagy, which plays a cyto-protective role that may impede their efficacy in cancer treatment. Moreover, they support anti-autophagy/RTK inhibitor combination therapy as an advanced approach for improving the efficacy of currently FDA (Food and Drug Administration) approved RTK inhibitors. This approach might provide significant benefits to patients with solid tumors in which at least one of the mentioned RTKs is deregulated. Thus, we conclude that it would be pertinent to conduct a more detailed examination of the use of this combination strategy (Fig. 1c) that has been proposed by different research groups.

## Conclusions

The idea that targeting RTKs might be adopted as a suitable strategy for the clinical management of cancer has persisted since the moment it became clear that dysregulated RTK function is a frequent event in tumor cells [65]. Currently, the efficacy and tolerability of most of the available RTK inhibitors are not sufficient, and therefore, their continuous improvement is urgently needed. The chief problem with the use of RTK inhibitors in clinical oncology is their targeting of multiple RTKs, which can evoke several side effects [66]. On the other hand, a large issue that compromises the wider use in oncology is the development of drug resistance in treated patients [67]. Lately, there have been numerous reports on the cyto-protective role of autophagy related to the efficacy of RTK inhibitors [41, 42]. A relatively high incidence of autophagy during treatment with these inhibitors suggests that autophagy is a probable cause of primary or acquired drug resistance. It also justifies the numerous ongoing preclinical and clinical studies that are considering autophagy inhibitors to improve anti-cancer therapy [68]. The autophagy inhibitors CQ and bafilomycin A1 are frequently mentioned in combination therapy with RTK inhibitors [45–48, 52, 58]. In in vitro and in vivo studies, abolishing autophagosome formation had additive or synergistic effects regarding the anti-tumor activity of RTK inhibitors [60, 69].

Currently, available preclinical and clinical data indicate that autophagy inhibitors, given in combination with RTK inhibitors, might be a promising approach for treating cancer patients with deregulated RTKs. This approach might ensure not only higher efficacy of these inhibitors but also fewer toxic side effects, particularly in those patients who become resistant during drug treatment. By highlighting the increased efficacy of combination approaches with respect to a single RTK-targeted strategy, we would like to express our belief that this line of attack might be worth of intense investigation in all malignancies where resistance to RTK inhibitors is a problem. We believe that inhibiting autophagy in conjunction with RTK inhibitor treatment could give a better chance to improve the battle against solid tumors. It remains to determine whether combined therapeutic strategy might be considered in the future clinical practice, even before the resistance to RTK inhibitors is developed.

## Abbreviations

RTK: receptor tyrosine kinase
CQ: chloroquine
3-MA: 3-methyladenine
ALK: anaplastic lymphoma kinase
EGFR: epidermal growth factor receptor
VEGFR: vascular endothelial growth factor receptor
PDGFR: platelet-derived growth factor receptor
c-Kit: stem cell tyrosine kinase receptor
NSCLC: non-small cell lung cancer
siRNA: small interfering RNA
MET (HGFR): hepatocyte growth factor receptor
